# IL-33/ST2 plays a critical role in endothelial cell activation and microglia-mediated neuroinflammation modulation

**DOI:** 10.1186/s12974-018-1169-6

**Published:** 2018-05-04

**Authors:** Kelei Cao, Xiang Liao, Jiahui Lu, Shu Yao, Fengjiao Wu, Xingxing Zhu, Dongyan Shi, Shuang Wen, Lixin Liu, Hong Zhou

**Affiliations:** 1 Department of Immunology, Nanjing Medical University, 101 Longmian Avenue, Nanjing, 211166 JS China; 2 Department of Immunology, Anhui Provincial Key Laboratory of Infection and Immunity, Bengbu Medical College, Bengbu, 233030 China; 3 Department of Pharmacology, College of Medicine, University of Saskatchewan, Saskatoon, Saskatchewan S7N 5E5 Canada

**Keywords:** CNS inflammation, IL-33/ST2, Neutrophil infiltration, Endothelial activation, Microglia activation, Intravital microscopy

## Abstract

**Background:**

Interleukin-33 (IL-33) is increasingly being recognized as a key immunomodulatory cytokine in many neurological diseases.

**Methods:**

In the present study, wild-type (WT) and IL-33^−/−^ mice received intracerebroventricular (i.c.v.) injection of lipopolysaccharide (LPS) to induce neuroinflammation. Intravital microscopy was employed to examine leukocyte–endothelial interactions in the brain vasculature. The degree of neutrophil infiltration was determined by myeloperoxidase (MPO) staining. Real-time PCR and western blotting were used to detect endothelial activation. Enzyme-linked immunosorbent assay and quantitative PCR were conducted to detect pro-inflammatory cytokine levels in the brain.

**Results:**

In IL-33^−/−^ mice, neutrophil infiltration in the brain cortex and leukocyte–endothelial cell interactions in the cerebral microvessels were significantly decreased as compared to WT mice after LPS injection. In addition, IL-33^−/−^ mice showed reduced activation of microglia and cerebral endothelial cells. In vitro results indicated that IL-33 directly activated cerebral endothelial cells and promoted pro-inflammatory cytokine production in LPS-stimulated microglia.

**Conclusions:**

Our study indicated that IL-33/ST2 signaling plays an important role in the activation of microglia and cerebral endothelial cells and, therefore, is essential in leukocyte recruitment in brain inflammation.

**Graphical abstract:**

The role of IL-33/ST2 in LPS induced neuroinflammation
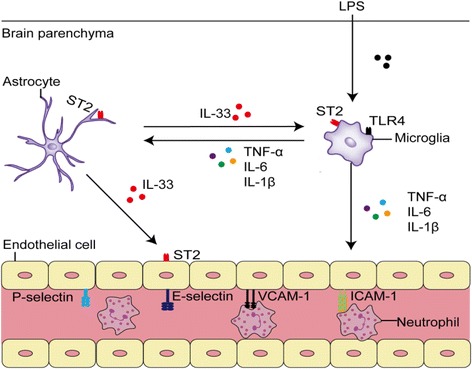

## Highlights

IL-33^−/−^ mice exhibit low inflammatory cytokine expression and microglial activation in LPS-induced neuroinflammation.

IL-33 mediates endothelial cell activation and neutrophil infiltration in the brain.

## Background

The brain is considered an immune-privileged organ [1, 2]. In response to various bacterial infections, robust innate immune responses can occur in the brain [3, 4]. Central nervous system (CNS) inflammatory conditions, such as bacterial meningitis [5, 6] and viral infections [7, 8], are generally associated with elevated innate immune responses, including activation of residential microglia and recruitment of immune cells from the circulation. Microglial cells are considered the immune defensive macrophages in the CNS [9] and play a dominant role in the innate immune response to infectious diseases in the CNS [10]. The neuroinflammatory response starts with microglial activation [11–13]. Pro-inflammatory cytokines produced by glial cells, such as tumor necrosis factor-α (TNF-α) [14], are widely recognized to activate endothelial cells and induce the expression of adhesion molecules [15–17], which mediate leukocyte-endothelial interactions and their subsequent transmigration across the blood-brain barrier (BBB) into the brain parenchyma [18–20].

Interleukin-33 (IL-33), a member of the IL-1 cytokine family, has attracted growing research interest. IL-33 was first described as a nuclear protein [21] but also exerts its cytokine activity by binding to a heteromeric receptor comprising ST2 and IL-1 receptor accessory protein (IL-1RAcp) [22, 23]. IL-33 is generally released by damaged cells to act as an alarmin [24, 25], and it is reported to be released from astrocytes or oligodendrocytes [26, 27]. It has an important effect on innate and adaptive immunity [25, 28, 29] and contributes to the maintenance of tissue homeostasis. IL-33 expression levels in the CNS are the highest among all organs [21]. Recently, there has been increased research interest in the role of IL-33 in the CNS. In a mouse model of traumatic brain injury, the deficiency of IL-33/ST2 suppressed microglia/macrophage infiltration in the injured region [30]. In a mouse model of CNS injury, IL-33 was critical in inducing chemokine production for monocyte recruitment through microglia [26]. In an animal model of stroke, the IL-33/ST2 axis functioned as an immuno-regulatory mechanism that enhanced M2 polarization of microglia and reduced stroke [27]. Together, these findings suggest that IL-33 signaling is important for inflammation modulation and immune cell recruitment in CNS diseases. However, the detailed mechanisms underlying the contribution of IL-33/ST2 signaling to neuroinflammation modulation and leukocyte recruitment remain unclear.

In this study, we employed intravital microscopy to examine the role of IL-33 in leukocyte recruitment during lipopolysaccharide (LPS)-induced neuroinflammation in mice, and we evaluated the effects of IL-33 on endothelial cells and microglia in vitro. In response to LPS administration, IL-33^−/−^ mice exhibited significantly lower expression of adhesion molecules, such as VCAM-1, ICAM-1, and P-selectin, than wild-type (WT) mice, suggesting that IL-33 contributes to brain endothelial cell activation. In addition, pro-inflammatory cytokine levels and microglial activation were lower in IL-33^−/−^ than in WT mice. IL-33 stimulated cerebral endothelial cell activation in vitro, and IL-33 was found to be essential to promote LPS-stimulated microglial pro-inflammatory cytokine production. Taken together, these results demonstrated that IL-33 plays an important role in endothelial cell activation and immunomodulation through microglia in neuroinflammation.

## Methods

### Animals and reagents

Male C57BL/6J mice (7−8 weeks old, 20−25 g), used as WT controls, were purchased from the Animal Core Facility of Nanjing Medical University. IL-33-deficient (IL-33^−/−^) mice (C57BL/6J background) were obtained from Dr. Hiroshi Kiyonari (Laboratory for Animal Resources and Genetic Engineering, Center for Developmental Biology, Institute of Physical and Chemical Research, Kobe, Japan). ST2-deficient (ST2^−/−^) mice (C57BL/6J background) were obtained from Dr. Fang Zheng (Department of Immunology, School of Basic Medicine, Tongji Medical College, Huazhong University of Science and Technology, Wuhan, China). All mice were maintained in the Animal Core Facility of Nanjing Medical University under specific pathogen-free conditions with free access to food and water. All animal experimental protocols were reviewed and approved by the Institutional Animal Care and Use Committee of Nanjing Medical University and were in compliance with institutional guidelines.

LPS (*Escherichia coli* serotype 0111: B4 strain) was purchased from InvivoGen (San Diego, CA, USA). Recombinant mouse IL-33 protein was purchased from R&D Systems (Minneapolis, MN, USA). Antibodies against VCAM-1, P-selectin, E-selectin, IL-33, myeloperoxidase (MPO), and mouse serum albumin were purchased from Abcam (Cambridge, MA, USA). Antibody against ST2 was purchased from Santa Cruz Biotechnology (Santa Cruz, CA, USA). Antibodies against β-actin, ERK, phospho-ERK, p38 mitogen-activated protein kinase (MAPK), phospho-p38 MAPK, JNK, phospho-JNK, NF-κB p65, and phospho-NF-κB p65 were purchased from Cell Signaling Technology (Beverly, MA, USA). The anti-ionized calcium-binding adaptor molecule 1 (Iba-1) antibody was purchased from Wako Pure Chemical (Osaka, Japan).

### Cell culture

The murine cerebral microvascular endothelial cell line bEND.3 and the murine microglial cell line BV2 were purchased from the American Type Culture Collection (Manassas, VA, USA). Cells were cultured in high-glucose Dulbecco’s modified Eagle’s medium (DMEM; GE Healthcare Hyclone, Logan, UT, USA) containing 10% fetal bovine serum (FBS; Gibco, Gaithersburg, MD, USA), at 37 °C in a 5% CO_2_ incubator. The cells were serum-starved for 12 h before they were stimulated with IL-33 for western blotting.

### Intracerebroventricular LPS injection

Mice were administered an intracerebroventricular (i.c.v.) LPS injection as previously described [10]. In brief, the mice were anesthetized via intraperitoneal injection of 200 mg/kg ketamine and 10 mg/kg xylazine. The mice were placed onto a rodent stereotaxic frame (David Kopf Instruments, Tujunga, CA, USA). Then, 2 μg of LPS in 2 μl saline was injected into the left ventricle using a Hamilton microsyringe over a 5-min period. Control animals received an i.c.v. injection of an equal volume of saline. After i.c.v. injection, the animals were maintained at 36 ± 1 °C on a thermostatic heating system (Harvard Apparatus, MA, USA) throughout the experiment.

### Intravital microscopy

Intravital microscopy was performed as previously described [10]. After anesthetization, a craniotomy was performed in the right parietal bone using a high-speed drill and the dura was carefully removed to expose the brain microvessels. The mice were given an intravenous injection of rhodamine 6G (Sigma-Aldrich, St. Louis, MD, USA) (0.5 mg/kg body weight) to label leukocytes. Leukocyte–endothelial interactions in the brain microvasculature were photographed using a sCMOS camera (ORCA-Flash 4.0; Hamamatsu, Japan) mounted on Nikon FN1 microscope. Three different microvessels with diameters of 30−60 μm were visualized and imaged. Rolling leukocytes were defined as cells moving at a velocity less than that of erythrocytes. Cells were considered adherent when they remained stationary for 30 s.

### Enzyme-linked immunosorbent assay (ELISA)

The mice were anesthetized after i.c.v. LPS injection and subsequently perfused through the heart with 20−30 ml of ice-cold PBS to clear blood cells and proteins from the circulation. The brains were rapidly removed and subsequently homogenized in 1 ml of ice-cold PBS, followed by centrifugation at 12,000×*g* for 5 min at 4 °C. The supernatants were assayed for TNF-α, IL-6, IL-1β, and MCP-1 concentrations using commercial ELISA kits (for TNF-α, IL-6, and IL-1β: BD Biosciences, San Diego, CA, USA; for MCP-1: R&D Systems) following the manufacturers’ instructions.

### RNA isolation and quantitative reverse transcription (qRT)-PCR

After perfusion of the heart with ice-cold PBS, the mouse brain was collected and homogenized in 1 ml of TRIzol (Takara Bio, Shiga, Japan) on ice, and RNA was extracted using TRIzol reagent following the protocols supplied by the manufacturer. One microgram of total RNA was reverse-transcribed into cDNA using PrimeScript RT Master Mix (Takara Bio). Then, real-time PCR was conducted using SYBR® Green (Bio-Rad Laboratories, Hercules, CA, USA) following the manufacturer’s instructions. The following primer sets were used: TNF-α fwd, 5′-ACGGCATGGATCTCAAAGAC-3′, and TNF-α rev, 3′-AGATAGCAAATCGGCTGACG-5′; IL-6 fwd, 5′-ACAACCACGGCCTTCCCTAC-3′, and IL-6 rev, 3′-AGATAGCAAATCGGCTGACG-5′; IL-1β fwd, 5′-TGTCTTGGCCGAGGACTAAGG-3′, and IL-1β rev, 3′-TGGGCTGGACTGTTTCTAATGC-5′; P-selectin fwd, 5′-TCCAGGAAGCTCTGACGTACTTG-3′, and P-selectin rev, 3′-GCAGCGTTAGTGAAGACTCCGTAT-5′; E-selectin fwd, 5′-TGAACTGAAGGGATCAAGAAGACT-3′, and E-selectin rev, 3′-GCCGAGGGACATCATCACAT-5′; ICAM-1 fwd, 5′-CCTGTTTCCTGCCTCTGAA-3′, and ICAM-1 rev, 3′-GTCTGCTGAGACCCCTCTTG-5′; VCAM-1 fwd, 5′-TGACAAGTCCCCATCGTTGA-3′, and VCAM-1 rev, 3′-ACCTCGCGACGGCATAATT-5′; IL-33 fwd 5′-TCCAACTCCAAGATTTCCCCG-3′, and IL-33 rev, 3′-AAGACGGTACAGATGACGTAC-5′; and GAPDH fwd, 5′-TGCAGTGGCAAAGTGGAGATT-3′, and GAPDH rev, 3′-TCGCTCCTGGAAGATGGTGAT-5′. The housekeeping gene *GAPDH* was used for normalization. qPCR were conducted in triplicate for each sample, and target mRNA levels were quantified using the 2^–ΔΔCt^ method.

### Western blotting

Mice were anesthetized and perfused through the heart with ice-cold PBS to clear blood cells and proteins from the circulation. The mouse brains were rapidly removed and homogenized in 1 ml of ice-cold PBS and then centrifuged at 13,800×*g* for 5 min at 4 °C. For cell-based assays, cells were treated with radioimmunoprecipitation assay lysis buffer for 30 min at 4 °C and centrifuged at 13,800×*g* for 10 min at 4 °C. The supernatants of brain homogenates or cell lysates were diluted in loading buffer and boiled at 100 °C for 10 min. The samples were subjected to 10% sodium dodecyl sulfate-polyacrylamide gel electrophoresis and the proteins were transferred to a polyvinylidene difluoride membrane (Millipore, Billerica, MA, USA). The membranes were blocked with 5% bovine serum albumin in PBS for 2 h at room temperature and then incubated with primary antibody overnight at 4 °C. The membranes were washed with PBST (0.05% Tween-20 in PBS) three times and then incubated with species-appropriate HRP-conjugated secondary antibody for 1.5−2 h at room temperature. Then, the membranes were washed with PBST three times and subjected to immunodetection with enhanced chemiluminescence reagents (PerkinElmer, Waltham, MA, USA).

### Primary culture of purified microglia and astrocytes

The brains of newborn mice were harvested and the cerebella, white matter, and leptomeninges were separated from the cerebral cortices. Then, the cerebral cortices were trypsinized for 5 min at 37 °C and filtrated through a 70-μm pore-size filter (Millipore, Billerica, MA, USA). Cells from six cerebra were seeded in a 75-cm^2^ culture flask containing 15 ml of DMEM/F12 with 10% FBS and incubated in 5% CO_2_ at 37 °C. After 24 h, the entire medium was replaced, and then half of the medium was exchanged every 3−4 days. On days 13−14, microglia were isolated from the mixed glial culture by shaking the flask at 200 rpm for 1 h at 37 °C [31]. The microglia were centrifuged and seeded in 24-well plates for further stimulation. The mixed glial cells were passaged two to three times and shaken at 220 rpm for 6 h; the supernatants were discarded and the remaining adherent cells were collected as astrocytes. The purity of the isolated microglia and astrocytes was 95 and 90%, respectively.

### Isolation and culture of murine cerebral endothelial cells

Seven- to 8-week-old mice were sacrificed, and their brains were collected. The cerebella, striata, optic nerves, and white matter were removed, and cerebral cortices were collected. The tissue samples were digested in 15 ml of 0.1% collagen B (Roche, Indianapolis, IN, USA) supplemented with 30 U/ml DNase I (Sigma, St. Louis, MO, USA) for 1.5 h at 37 °C and shaken every half hour. The microvessel pellets were resuspended in medium supplemented with 3 ng/ml of bovine fibroblast growth factor (Peprotech, Rocky Hill, NJ, USA), 30% FBS, 10 U/ml of heparin, 100 U/ml of penicillin, and 100 μg/ml of streptomycin. The microvessel suspensions were placed in six-well plates pre-coated with rat-tail collagen I (Sigma-Aldrich) and incubated in 5% CO_2_ at 37 °C. The medium was changed within the first 24 h and then every 2 days. The endothelial cells grew to confluence within 7–10 days.

### Immunohistochemical analysis

Brain blocks were embedded in paraffin after fixation in 4% paraformaldehyde and then sliced into 4-μm sections. After blocking with bovine serum albumin, for the detection of infiltrating neutrophils, the sections were incubated with anti-MPO antibodies at 4 °C overnight. This was followed by a 1-h incubation with horseradish peroxidase (HRP)-labeled secondary antibodies at room temperature. Different fields of view were analyzed at magnifications of ×100 and ×400, and cells stained positive for primary antibody were counted under a Nikon E100 microscope.

### Immunofluorescence

Mice were perfused with ice-cold PBS and 4% paraformaldehyde, and the brains were collected, conserved in paraformaldehyde, and dehydrated in a 30% sucrose solution. Then, 25-μm cortex sections were incubated overnight with anti-Iba1. Iba-1 is known to be the activation marker of microglia. The cortex was observed in four fields of view at a primary magnification of ×200 in every brain section. Images were captured using a fluorescence microscope (Nikon Eclipse TE2000-S, Nikon, Tokyo, Japan) at a magnification of ×200.

### Statistical analysis

Statistical analysis was performed using GraphPad Prism 5 software. The data are shown as the mean ± standard error of the mean (SEM). Means were compared using Student’s *t* test for two groups or one-way ANOVA for multiple groups. *P* < 0.05 was considered significant.

## Results

### IL-33 and ST2 expression in the brain after i.c.v. LPS injection

**Fig. 1:**
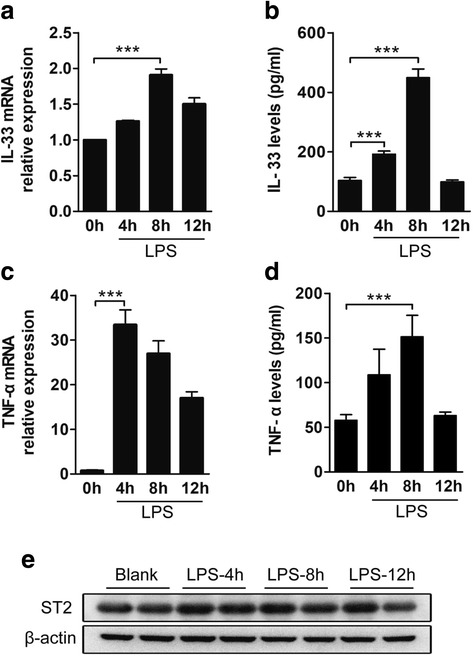
IL-33 and ST2 expression in the brain after LPS stimulation. I.c.v. LPS injection, used as a standard neuroinflammation model, resulted in a significant increase in inflammatory cytokines in WT mice. IL-33 (**a**, **b**) and TNF-α (**c**, **d**) expression and concentrations in the brains of WT mice were detected via real-time quantitative PCR (**a**, **c**) and ELISA (**b**, **d**) at 4, 8, and 12 h after LPS injection. **e** ST2 expression in the brains of WT mice was measured by western blot analysis (*n* = 2). The results in **a**−**d** are presented as the mean ± SEM and are representative of three independent experiments, *n* ≥ 5 mice for all treatment groups; *P < 0.05, **P < 0.01, and ***P < 0.001 compared to the untreated group (0 h)

i.c.v. injection of LPS induced strong expression of inflammatory cytokines in the brains of WT mice. The mRNA (Fig. 1a) and protein (Fig. 1b) levels of IL-33 were gradually increased at 4 h and peaked at 8 h after i.c.v. LPS injection and decreased thereafter. The mRNA levels of TNF-α peaked at 4 h (Fig. 1c), the protein levels of TNF-α peaked at 8 h, and both were then decreased (Fig. 1c, d). Surprisingly, the protein levels of ST2 remained unchanged from 4 to 12 h after LPS injection (Fig. 1e).

### IL-33 deficiency decreases LPS-induced neutrophil recruitment into the brain

**Fig. 2:**
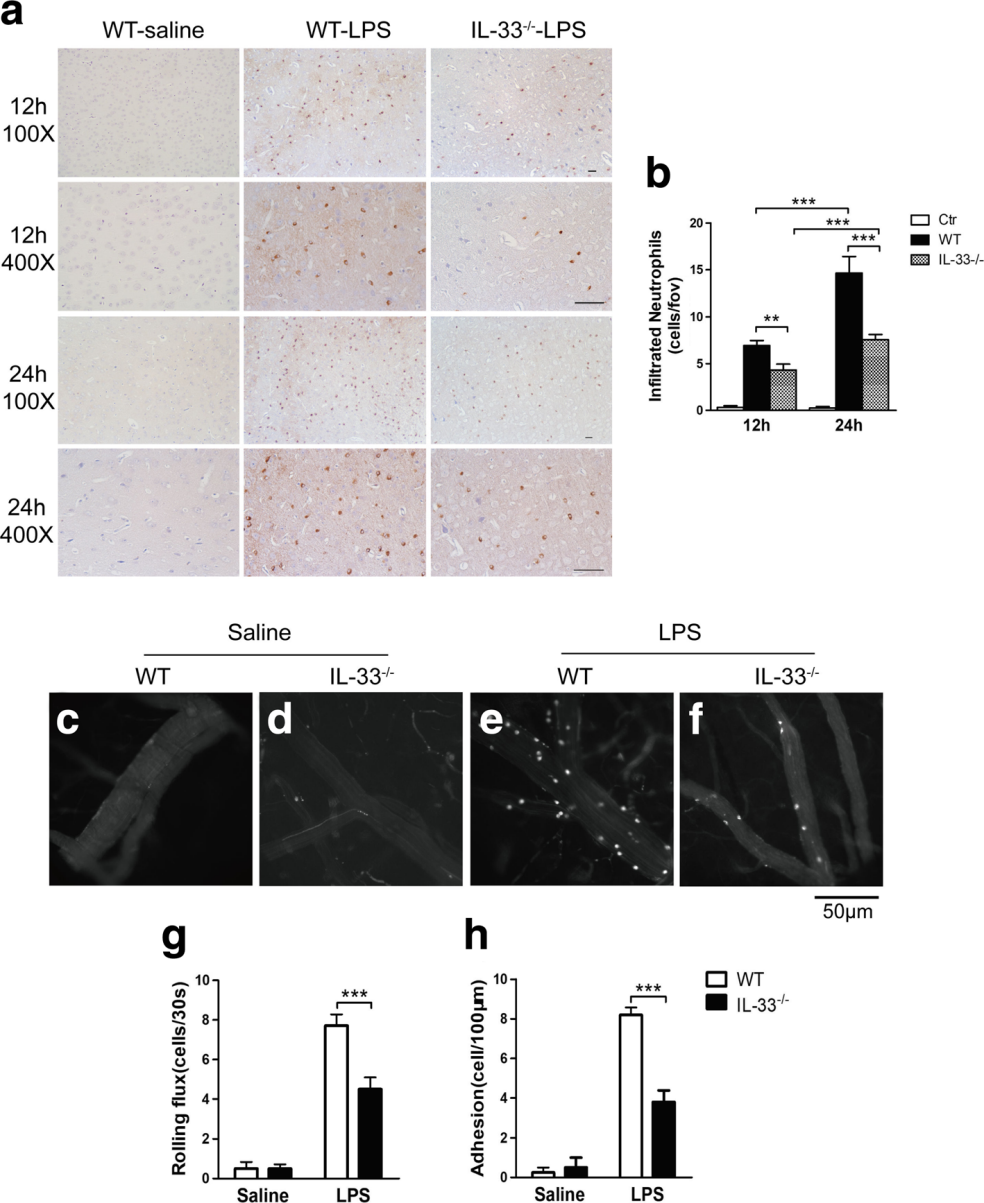
IL-33^−/−^ mice exhibit decreased neutrophil recruitment and leukocyte–endothelial interactions in brain microvessels after i.c.v. LPS administration. WT and IL-33^−/−^ mice received i.c.v. saline or LPS administration, and after 12 or 24 h, MPO staining was performed to quantitate the neutrophils infiltrating the cortex (**a**). IL-33^−/−^ mice exhibited significantly reduced MPO-positive neutrophils in the cortex (**a**, **b**). At 4 h after LPS injection, intravital microscopy was employed to visualize leukocyte–endothelial interactions. WT and IL-33^−/−^ mice were treated with saline (**c**, **d**) or LPS (**e**, **f**). Quantitative data of infiltrated neutrophils (**b**) and leukocyte rolling (**g**) and adhesion (**h**) are shown as the mean ± SEM. *n* = 4 mice per group. **P < 0.01 and ***P < 0.001

To examine the role of IL-33 in neutrophil recruitment in brain inflammation, we administered i.c.v. LPS injection to WT and IL-33^−/−^ mice. Brain sections from the mice were stained with MPO to evaluate neutrophil recruitment. Neutrophil infiltration into the brain started at 12 h (Fig. 2a) and peaked at 24 h after LPS injection (Fig. 2a). Compared with WT mice, IL-33^−/−^ mice exhibited significantly reduced neutrophil infiltration (Fig. 2b). We used intravital microscopy to examine the role of IL-33 in leukocyte endothelial interactions in brain microvessels. i.c.v. injection of saline did not induce leukocyte rolling or adhesion in brain microvessels of WT (Fig. 2c) and IL-33^−/−^ mice (Fig. 2d). In WT mice, i.c.v. LPS administration induced substantial leukocyte–endothelial interactions in brain microvessels (Fig. 2e). Interestingly, i.c.v. LPS-elicited leukocyte–endothelial cell interactions were significantly affected by the deficiency of IL-33 (Fig. 2f): in IL-33^−/−^ mice, leukocyte rolling (Fig. 2g) and adhesion (Fig. 2h) induced by i.c.v. LPS were significantly reduced as compared to those in WT mice.

### IL-33 deficiency decreases brain endothelial adhesion molecule expression but does not affect blood–brain barrier (BBB) permeability

**Fig. 3:**
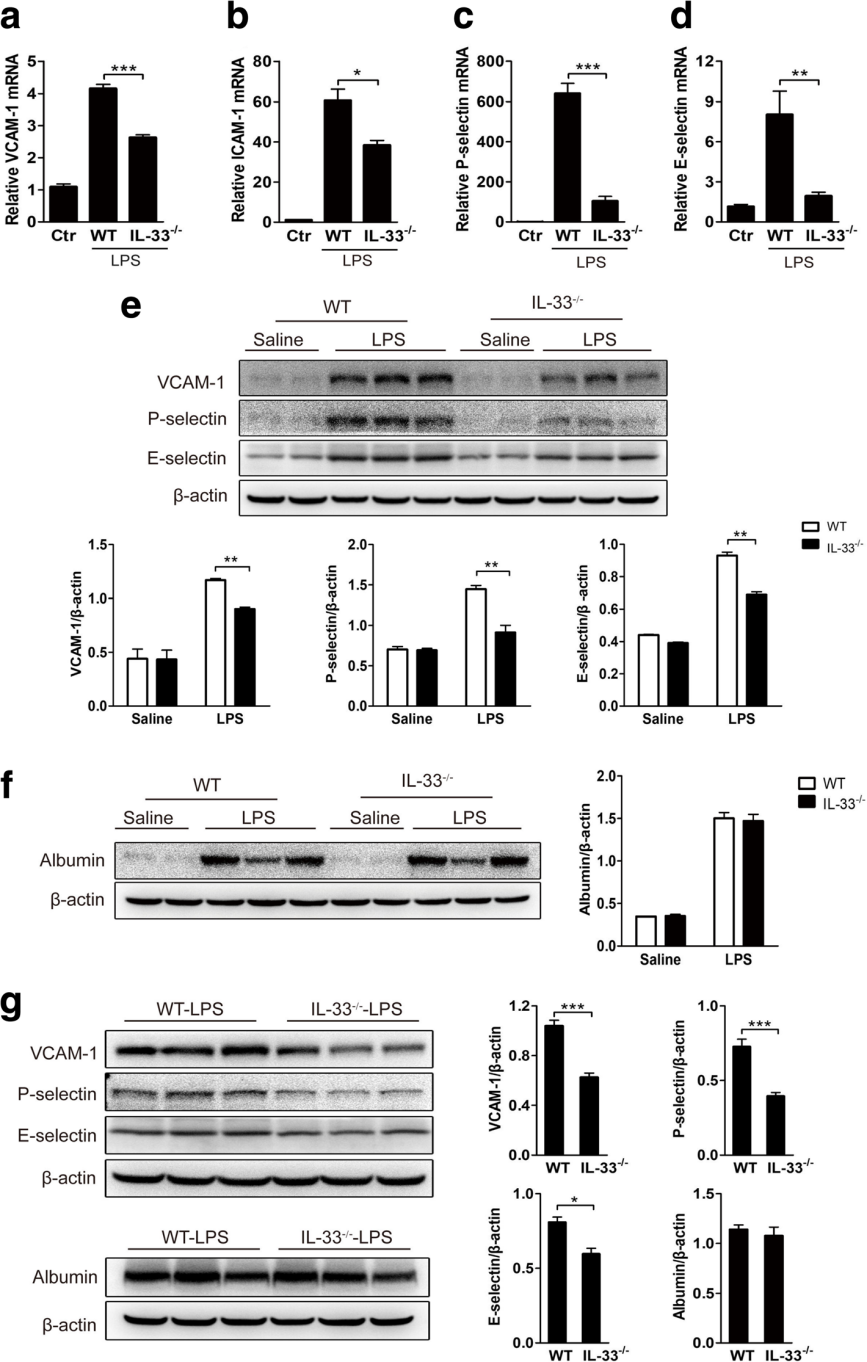
Role of IL-33 in brain endothelial adhesion molecule expression and blood–brain barrier (BBB) integrity changes. Four hours after i.c.v. LPS injection, the mRNA levels of adhesion molecules in WT and IL-33^−/−^ mouse brains were measured by qRT-PCR. The mRNA levels of VCAM-1 (**a**), ICAM-1 (**b**), P-selectin (**c**), and E-selectin (**d**) were decreased in IL-33^−/−^ mice. The VCAM-1, P-selectin, and E-selectin protein levels, as measured by western blotting, were significantly decreased at 4-h i.c.v. LPS treatment in IL-33^−/−^ mice compared to WT mice (**e**). Western blot analysis of brain albumin levels was performed to examine BBB integrity between WT and IL-33^−/−^ mice after 4-h LPS i.c.v. injection (**f**). At 24 h after LPS injection, western blot analysis of VCAM-1, P-selectin, E-selectin, and albumin expression (**g**) revealed that VCAM-1, P-selectin, and E-selectin were all decreased, but no change in albumin level was observed in IL-33^−/−^ as compared to WT mice (**g**). *n* = 4 mice per group; *P < 0.05, **P < 0.01, and ***P < 0.001

To examine whether IL-33 deficiency affects cerebral endothelial activation, we determined the mRNA levels of the endothelial adhesion molecules VCAM-1, ICAM-1, P-selectin, and E-selectin in the brains of IL-33^−/−^ mice by qRT-PCR. Four hours after i.c.v. LPS administration, the mRNA expression of all these adhesion molecules was significantly elevated in LPS-treated brains of WT mice (Fig. 3a–d). Interestingly, in IL-33^−/−^ mice, mRNA expression of these adhesion molecules was significantly lower than in WT mice. In addition, VCAM-1, P-selectin, and E-selectin protein levels were lower in IL-33^−/−^ than in WT mice in 4-h LPS-induced neuroinflammation (Fig. 3e). BBB permeability can affect leukocyte recruitment in the brain. To determine whether IL-33 deficiency led to altered BBB permeability, we measured the brain albumin leaked from the circulation. Interestingly, the BBB permeability showed no difference between WT and IL-33^−/−^ mice treated with i.c.v. LPS (Fig. 3f). In addition, at 24 h after LPS injection, VCAM-1, P-selectin, and E-selectin protein levels were also lower in IL-33^−/−^ than in WT mice, and albumin protein levels in the brains showed no difference between IL-33^−/−^ and WT mice (Fig. 3f). Thus, IL-33 contributes to cerebral endothelial activation through upregulating the expression of endothelial adhesion molecules but may not play a role in the breakage of the BBB during LPS-induced neuroinflammation.

### IL-33 mediates cerebral endothelial cell activation

**Fig. 4:**
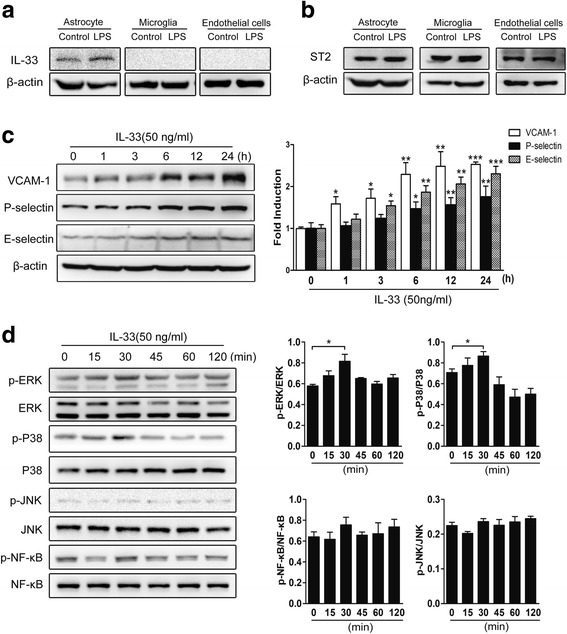
IL-33 stimulates endothelial cell activation and adhesion molecule expression. Primary astrocytes, microglia, and endothelial cells were isolated from WT mice and treated with saline or LPS (100 ng/ml) for 4 h, and then, IL-33 (**a**) and ST2 (**b**) expression were measured by western blotting. Brain bEnd.3 endothelial cells were stimulated with IL-33 (50 ng/ml) for 0, 1, 3, 6, 12, and 24 h, and VCAM-1, P-selectin, and E-selectin (**c**) expression were detected by western blotting. **d** Brain endothelial bEnd.3 cells were incubated with IL-33 (50 ng/ml) for the indicated time periods, and phosphorylated and total ERK, p38, JNK, and NF-κB p65 in cell lysates were detected by using western blotting. *n* = 4 mice for each group. Data are shown as the mean ± SEM; *P < 0.05, **P < 0.01, and ***P < 0.001

To determine the role of IL-33 in brain endothelial cell activation, we stimulated brain primary endothelial cells with LPS and examined the expression of IL-33 and ST2 in these cells. In addition, we examined the expression of IL-33 and ST2 in LPS-stimulated astrocytes and microglia. After LPS treatment for 4 h, increased IL-33 expression was observed only in cultured primary astrocytes (Fig. 4a). IL-33 expression was not detectable in microglia and endothelial cells with or without LPS treatment (Fig. 4a). ST2 expression was noted in all three cell types and remained unchanged with LPS administration (Fig. 4b). To determine the role of IL-33 in endothelial ST2 receptor expression, we treated bEnd.3 brain endothelial cells with IL-33 (50 ng/ml) for 0, 1, 3, 6, 12, and 24 h. The protein levels of VCAM-1, P-selectin, and E-selectin in these cells were increased over time and became significant at 1−24, 6−24, and 3−24 h, respectively, after IL-33 stimulation (Fig. 4c). IL-33 treatment also activated multiple signal molecules in brain endothelial cells. After 30 min of stimulation, increased levels of phospho-ERK and phospho-p38 MAPK were observed in IL-33-treated bEnd.3 cells (Fig. 4d). However, no significant changes in the phosphorylation levels of JNK and NF-κB p-65 were noted.

### IL-33 deficiency decreases neuroinflammation and microglial activation

**Fig. 5:**
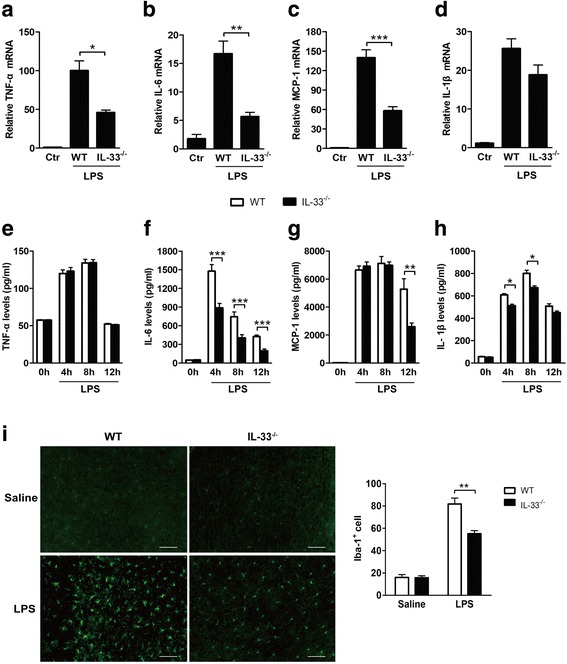
Pro-inflammatory cytokine levels and microglial activation in the brains of WT and IL-33^−/−^ mice after i.c.v. LPS injection. WT mice and IL-33^−/−^ mice received i.c.v. injection of saline or LPS, and pro-inflammatory cytokine expression levels were detected in the brains by using qRT-PCR and ELISAs. The mRNA levels of the pro-inflammatory cytokines TNF-α (**a**), IL-6 (**b**), and MCP-1 (**c**) were significantly decreased in IL-33^−/−^ mice 4 h after LPS administration, and no differences in IL-1β levels (**d**) were observed in the brains of LPS-treated WT and IL-33^−/−^ mice. TNF-α production in the brains did not differ between LPS-treated WT and IL-33^−/−^ mice (**e**), but IL-33^−/−^ mice produced significantly less IL-6 (**f**), MCP-1 (**g**), and IL-1β (**h**) in the brains than WT mice. Twelve hours after i.c.v. LPS injection, cortex sections of WT and IL-33^−/−^ mice were immunostained with anti-Iba1 antibody to determine microglial activation, and IL-33^−/−^ mice exhibited significantly decreased microglial activation as compared to WT mice (**i**). Data are presented as the mean ± SEM, with *n* = 5 for all groups; *P < 0.05, **P < 0.01, and ***P < 0.001

In acute CNS inflammation, cytokines secreted from glial cells play an essential role in endothelial activation and leukocyte recruitment [15]. At 4 h after i.c.v. LPS injection, the mRNA levels of pro-inflammatory cytokines TNF-α, IL-6, MCP-1, and IL-1β were significantly elevated in the brains of WT mice (Fig. 5a–d). Compared to WT mice, IL-33^−/−^ mice exhibited lower mRNA levels of TNF-α, IL-6, and MCP-1, but not IL-1β in the brains (Fig. 5a–d). At the protein level, TNF-α was not affected by IL-33 deficiency at 4, 8, and 12 h after LPS administration, whereas IL-6, MCP-1, and IL-1β were lower in IL-33^−/−^ than in WT mouse brains (Fig. 5e–h). Iba-1 is considered as the activation marker of microglia [32, 33]. Immunostaining with anti-Iba1 showed that LPS-treated IL-33^−/−^ mice had substantially lower numbers of activated microglial cells in cortex sections than LPS-injected WT mice (Fig. 5i). Therefore, deficiency of IL-33 affected microglial activation and inflammatory cytokine expression in LPS-induced brain inflammation.

### IL-33 promotes LPS-induced pro-inflammatory cytokine production in microglia

**Fig. 6:**
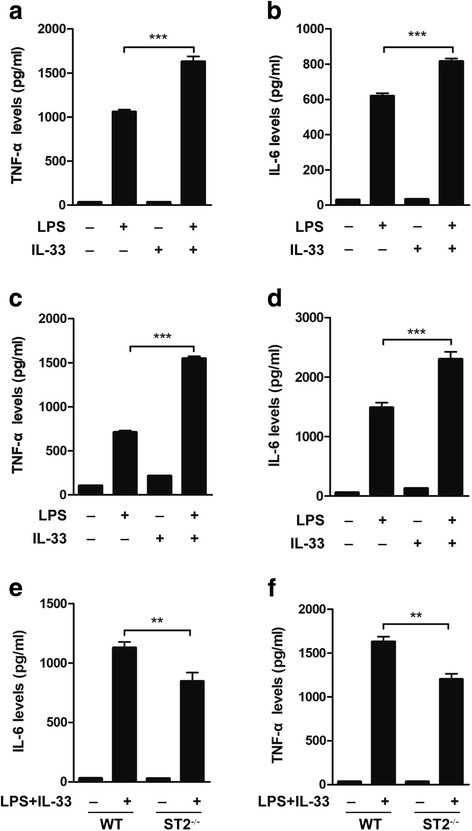
IL-33 promotes LPS-induced pro-inflammatory cytokine production by microglia. Primary microglia from WT mice were stimulated with LPS (100 ng/ml), IL-33 (50 ng/ml), or LPS (100 ng/ml) plus IL-33 (50 ng/ml), and after 4 h, the levels of the pro-inflammatory cytokines TNF-α (**a**) and IL-6 (**b**) in cell supernatants were measured by ELISA. Murine microglial BV2 cells were stimulated with LPS (100 ng/ml), IL-33 (50 ng/ml), or LPS (100 ng/ml) plus IL-33 (50 ng/ml) for 4 h, and the levels of TNF-α (**c**) and IL-6 (**d**) in supernatants were detected by ELISA. Primary microglia isolated from WT and ST2^−/−^ mice were stimulated with combined LPS (100 ng/ml) and IL-33 (50 ng/ml) for 4 h, and the levels of the TNF-α (**e**) and IL-6 (**f**) were detected in supernatants by ELISA. Data are shown as the mean ± SEM (*n* = 4 for all groups). **P < 0.01 and ***P < 0.001

To further confirm the role of IL-33 in microglial activation and pro-inflammatory cytokine production, we treated primary microglia with LPS (100 ng/ml) and/or IL-33 (50 ng/ml) for 4 h and measured the pro-inflammatory cytokine levels in the supernatant by ELISA. The TNF-α and IL-6 levels in the supernatants of cells treated with both LPS and IL-33 were significantly higher than those in the supernatants of cells treated with 4-h IL-33 or LPS alone (Fig. 6a, b). Similarly, treatment of murine BV2 microglial cells with LPS (100 ng/ml) and IL-33 (50 ng/ml) for simultaneously 4 h induced substantially higher levels of TNF-α and IL-6 in the culture supernatants than treatment with LPS or IL-33 alone (Fig. 6c, d). To determine whether the proinflammatory role of IL-33 is mediated through the receptor ST2, we cultured primary microglia from WT and ST2^−/−^ mice and treated them with LPS (100 ng/ml) plus IL-33 (50 ng/ml). In LPS + IL-33 treatment culture, the levels of TNF-α and IL-6 in the supernatant were significantly increased in the culture of WT microglia, and this response was significantly reduced in the supernatant of ST2^−/−^ microglial culture compared with the supernatant of WT microglial culture (Fig. 6e, f).

## Discussion

Recent studies have shown that IL-33, as a pleiotropic cytokine, is highly expressed in the brain [34]. While the function of IL-33 in the brain remains controversial [35], most studies have demonstrated that IL-33 released from astrocytes serves as a negative regulator of the progression of different types of CNS inflammation [26, 36, 37]. It has recently been reported that IL-33 exerts protective effects against hemorrhage and acute ischemic brain injury in animal models [26, 27]. In addition, Fu et al. have shown that IL-33 ameliorated cognitive decline in an animal model of Alzheimer’s disease [38]. In the present study, we showed that IL-33 mRNA and protein levels were significantly increased in the brain in response to i.c.v. LPS injection, and IL-33 was solely produced by astrocytes rather than endothelial cells. Further, IL-33 derived from astrocytes not only enhanced the inflammatory response of microglia to LPS but also played an essential role in mediating cerebral endothelial activation and subsequent leukocyte recruitment in LPS-induced brain inflammation. The role of IL-33 in the LPS injection model is partly different from that in other CNS inflammation models, which might lead to different phenomena. LPS injection can directly activate the TLR4 signal pathway and induce acute neuroinflammation, while in other models, inflammation is generally induced by hypoxia, toxic materials, or virus. In addition, IL-33 can directly trigger an increase in the expression of LPS receptor components (MD2/CD14 and TLR-4) and the MyD88 adaptor molecule. While these factors may explain the discrepant findings, the detailed mechanism underlying the difference requires further research.

Microglial activation is essential in all types of neuroinflammation [13]. Our results clearly showed that the IL-33 receptor ST2 is constitutively expressed in glial cells. Thus, we hypothesized that ST2 stimulation by its natural ligand IL-33 could promote microglial activation in response to LPS. Therefore, we assessed the role of IL-33 in LPS-induced microglial activation. Our data clearly showed that IL-33 deficiency resulted in significant reduction in inflammatory cytokine expression and microglial cell activation in the brain cortex. Interestingly, the inflammatory cytokine protein levels were not in accordance with the transcript levels. At the protein level, the differences in IL-1β were very small, and the difference in MCP-1 expression was apparent only at the last time point. We speculate that the difference in IL-1β protein was small because there was no significant difference in IL-1β transcript levels between WT and IL-33-deficient mice. In addition, the transcript level of MCP-1 was detected at 4 h, after transcription and translation, and the difference may appear later, until 12 h*.* IL-33 exerted a synergistic effect on glial activation with LPS and triggered an increase in inflammatory cytokine expression; however, this proinflammatory function was significantly decreased in microglia lacking ST2. These results indicated that IL-33 promotes microglial activation by LPS through ST2. Nevertheless, more detailed signaling mechanisms underlying the proinflammatory role of IL-33 warrant further investigation.

Upon i.c.v. LPS injection, IL-33^−/−^ mice had reduced leukocyte recruitment and neutrophil infiltration in brain parenchyma as compared to WT mice. In our study, the deficiency of IL-33 directly caused a significant reduction in rolling and adherent leukocyte numbers in cerebral microvessels, indicating that endothelial activation was compromised. Accordingly, the expression of adhesion molecules, such as VCAM-1, ICAM-1, P-selectin, and E-selectin, which facilitate leukocyte rolling and adherence in brain microvasculature, were significantly decreased in IL-33^−/−^ mice. At 4 and 24 h after LPS injection, compared to WT mice, IL-33^−/−^ mice did not show any difference in BBB permeability. Therefore, the lower neutrophil infiltration was not due to a change in BBB integrity but because of reduced endothelial activation. These findings prompted us to further explore the role of IL-33/ST2 in LPS-induced endothelial cell activation. Additionally, we show that ST2 was expressed in endothelial cells and IL-33 directly induced the expression of VCAM-1, P-selectin, and E-selectin as well as phosphorylation of ERK and p38 MAPK in cerebral endothelial cells. Obviously, IL-33 is an important endothelial activator in LPS-induced neuroinflammation.

As ST2 is expressed on endothelial cells and microglia, it is possible that IL-33 mediated neuroinflammatory modulation by affecting endothelial cell and microglial activation. IL-33^−/−^ mice had reduced inflammatory cytokine levels. However, the key inflammatory cytokine of endothelial activation, TNF-α, did not exhibit significant changes. Therefore, the reduced leukocyte recruitment in IL-33^−/−^ mice is unlikely to be caused by compromised microglial activation. Moreover, IL-33 can directly activate cerebral endothelial cells and upregulate the expression of endothelial cell adhesion molecules in vitro suggesting that IL-33 plays a key role in stimulating endothelial cell activation and leukocyte recruitment in the CNS.

## Conclusions

IL-33 is inducible in CNS astrocytes by LPS. Upon induction, IL-33 is able to boost the innate immune response by promoting microglial activation and activating endothelial cells to facilitate leukocyte recruitment. Our study unraveled a novel mechanism of the proinflammatory role of IL-33 in cerebral endothelial activation and leukocyte recruitment in neuroinflammation suggesting an integral role for IL-33 in CNS innate immunity.

## Abbreviations

CNS: Central nervous system
ELISA: Enzyme-linked immunosorbent assay
FBS: Fetal bovine serum
i.c.v.: Intracerebroventricular
ICAM-1: Intercellular cell adhesion molecule-1
IL-1β: Interleukin-1β
IL-33: Interleukin-33
IL-6: Interleukin-6
LPS: Lipopolysaccharide
MCP-1: Monocyte chemotactic protein-1
NF-κB: Nuclear factor-κB
PBS: Phosphate-buffered saline
TNF-α: Tumor necrosis factor-α
VCAM-1: Vascular cell adhesion molecule-1

## References

[1] Carson MJ, Doose JM, Melchior B, Schmid CD, Ploix CC (2006). CNS immune privilege: hiding in plain sight. Immunol Rev.

[2] Niederkorn JY (2006). See no evil, hear no evil, do no evil: the lessons of immune privilege. Nat Immunol.

[3] Böttcher T, von Mering M, Ebert S, Meyding-Lamadé U, Kuhnt U, Gerber J (2003). Differential regulation of toll-like receptor mRNAs in experimental murine central nervous system infections. Neurosci Lett.

[4] McKimmie CS, Fazakerley JK (2005). In response to pathogens, glial cells dynamically and differentially regulate toll-like receptor gene expression. J Neuroimmunol.

[5] Merres J, Hoss J, Albrecht LJ, Kress E, Soehnlein O, Jansen S (2014). Role of the cathelicidin-related antimicrobial peptide in inflammation and mortality in a mouse model of bacterial meningitis. J Innate Immun.

[6] Braun BJ, Slowik A, Leib SL, Lucius R, Varoga D, Wruck CJ (2011). The formyl peptide receptor like-1 and scavenger receptor MARCO are involved in glial cell activation in bacterial meningitis. J Neuroinflammation.

[7] Mutnal MB, Hu S, Schachtele SJ, Lokensgard JR (2014). Infiltrating regulatory B cells control neuroinflammation following viral brain infection. J Immunol.

[8] Durrant DM, Daniels BP, Pasieka T, Dorsey D, Klein RS (2015). CCR5 limits cortical viral loads during West Nile virus infection of the central nervous system. J Neuroinflammation.

[9] Hanisch UK, Kettenmann H (2007). Microglia: active sensor and versatile effector cells in the normal and pathologic brain. Nat Neurosci.

[10] Zhou H, Lapointe BM, Clark SR, Zbytnuik L, Kubes P (2006). A requirement for microglial TLR4 in leukocyte recruitment into brain in response to lipopolysaccharide. J Immunol.

[11] Prinz M, Priller J (2014). Microglia and brain macrophages in the molecular age: from origin to neuropsychiatric disease. Nat Rev Neurosci.

[12] Colonna M, Butovsky O (2017). Microglia function in the central nervous system during health and neurodegeneration. Annu Rev Immunol.

[13] Li Q, Barres BA. Microglia and macrophages in brain homeostasis and disease. Nat Rev Immunol. 2017; 10.1038/nri.2017.125. 10.1038/nri.2017.125 29151590

[14] Zhou H, Andonegui G, Wong CH, Kubes P (2009). Role of endothelial TLR4 for neutrophil recruitment into central nervous system microvessels in systemic inflammation. J Immunol.

[15] Wu F, Zhao Y, Jiao T, Shi D, Zhu X, Zhang M (2015). CXCR2 is essential for cerebral endothelial activation and leukocyte recruitment during neuroinflammation. J Neuroinflammation.

[16] Merrill JE, Benveniste EN (1996). Cytokines in inflammatory brain lesions: helpful and harmful. Trends Neurosci.

[17] Rao RM, Yang L, Garcia-Cardena G, Luscinskas FW (2007). Endothelial-dependent mechanisms of leukocyte recruitment to the vascular wall. Circ Res.

[18] Simmons SB, Liggitt D, Goverman JM (2014). Cytokine-regulated neutrophil recruitment is required for brain but not spinal cord inflammation during experimental autoimmune encephalomyelitis. J Immunol.

[19] Mantovani A, Cassatella MA, Costantini C, Jaillon S (2011). Neutrophils in the activation and regulation of innate and adaptive immunity. Nat Rev Immunol.

[20] D'Mello C, Le T, Swain MG (2009). Cerebral microglia recruit monocytes into the brain in response to tumor necrosis factoralpha signaling during peripheral organ inflammation. J Neurosci.

[21] Schmitz J, Owyang A, Oldham E, Song Y, Murphy E, McClanahan TK (2005). IL-33, an interleukin-1-like cytokine that signals via the IL-1 receptor-related protein ST2 and induces T helper type 2-associated cytokines. Immunity.

[22] Ali S, Huber M, Kollewe C, Bischoff SC, Falk W, Martin MU (2007). IL-1 receptor accessory protein is essential for IL-33-induced activation of T lymphocytes and mast cells. Proc Natl Acad Sci U S A.

[23] Chackerian AA, Oldham ER, Murphy EE, Schmitz J, Pflanz S, Kastelein RA (2007). IL-1 receptor accessory protein and ST2 comprise the IL-33 receptor complex. J Immunol.

[24] Cayrol C, Girard JP (2014). IL-33: an alarmin cytokine with crucial roles in innate immunity, inflammation and allergy. Curr Opin Immunol.

[25] Martin NT, Martin MU (2016). Interleukin 33 is a guardian of barriers and a local alarmin. Nat Immunol.

[26] Gadani SP, Walsh JT, Smirnov I, Zheng J, Kipnis J (2015). The glia-derived alarmin IL-33 orchestrates the immune response and promotes recovery following CNS injury. Neuron.

[27] Yang Y, Liu H, Zhang H, Ye Q, Wang J (2017). ST2/IL-33-dependent microglial response limits acute ischemic brain injury. J Neurosci.

[28] Oboki K, Ohno T, Kajiwara N, Arae K, Morita H, Ishii A (2010). IL-33 is a crucial amplifier of innate rather than acquired immunity. Proc Natl Acad Sci U S A.

[29] Kamijo S, Takeda H, Tokura T, Suzuki M, Inui K, Hara M (2013). IL-33-mediated innate response and adaptive immune cells contribute to maximum responses of protease allergen-induced allergic airway inflammation. J Immunol.

[30] Wicher G, Wallenquist U, Lei Y, Enoksson M, Li X, Fuchs B (2017). Interleukin-33 promotes recruitment of microglia/macrophages in response to traumatic brain injury. J Neurotrauma.

[31] Floden AM, Combs CK (2007). Microglia repetitively isolated from in vitro mixed glial cultures retain their initial phenotype. J Neurosci Methods.

[32] Fernandez-Calle R, Vicente-Rodriguez M, Gramage E, Pita J, Perez-Garcia C, Ferrer-Alcon M (2017). Pleiotrophin regulates microglia-mediated neuroinflammation. J Neuroinflammation.

[33] Wu DC, Jackson-Lewis V, Vila M, Tieu K, Teismann P (2002). Blockade of microglial activation is neuroprotective in the 1-methyl-4-phenyl-1,2,3,6-tetrahydropyridine mouse model of Parkinson disease. J Neurosci.

[34] Yasuoka S, Kawanokuchi J, Parajuli B, Jin S, Doi Y, Noda M, Sonobe Y, Takeuchi H, Mizuno T, Suzumura A (2011). Production and functions of IL-33 in the central nervous system. Brain Res.

[35] Du LX, Wang YQ, Hua GQ, Mi WL (2017). IL-33/ST2 pathway as a rational therapeutic target for CNS diseases. Neuroscience.

[36] Luo Y, Zhou Y, Xiao W, Liang Z, Dai J, Weng X (2015). Interleukin-33 ameliorates ischemic brain injury in experimental stroke through promoting Th2 response and suppressing Th17 response. Brain Res.

[37] Korhonen P, Kanninen KM, Lehtonen S, Lemarchant S, Puttonen KA, Oksanen M (2015). Immunomodulation by interleukin-33 is protective in stroke through modulation of inflammation. Brain Behav Immun.

[38] Fu AK, Hung KW, Yuen MY, Zhou X, Mak DS, Chan IC (2016). IL-33 ameliorates Alzheimer's disease-like pathology and cognitive decline. Proc Natl Acad Sci U S A.

